# Optimization of tamoxifen-induced gene regulation in cardiovascular research

**DOI:** 10.20517/jca.2022.12

**Published:** 2022-03-30

**Authors:** Abitha Sukumaran, Sakthivel Sadayappan

**Affiliations:** 1 Division of Oncology, Cincinnati Children’s Hospital Medical Center, University of Cincinnati College of Medicine, Cincinnati, OH 45229, USA.; 2 Heart, Lung and Vascular Institute, Division of Cardiovascular Health and Disease, Department of Internal Medicine, University of Cincinnati College of Medicine, Cincinnati, OH 45267, USA.

**Keywords:** Cardiomyopathy, heart failure, gene regulation

In this issue of *The Journal of Cardiovascular Aging*, Rouhi *et al.*^[1]^ have determined the effects of tamoxifen (TAM) and MerCreMer on cardiomyocyte transcriptome, cardiac function, and histopathology at an early developmental stage in mice.

The Cre-loxP system is a powerful and versatile tool to control site-specific recombination of mammalian genomic DNA. Site-specific Cre recombinase-mediated DNA recombination allows for the conditional control of gene expression within transgenic animals in a tissue-specific manner by employing a promoter known to be expressed specifically in such tissue of interest. More specifically, the gene of interest is flanked (floxed) by two loxP (locus of x-over, P1) sites in the presence of Cre recombinase, which then catalyzes the site-specific recombination of DNA between those loxP sites, leading to tissue-specific gene editing. However, the approach is flawed by the lack of control over the timing of Cre recombinase expression which often parallels the expression of the chosen promoter. Consequently, Cre-mediated, tissue-specific gene ablation could lead to embryonic, fetal, or neonatal lethality. Since the heart is the first organ to develop and start functioning during embryogenesis, the noted risks rule out examining gene function at later developmental stages. To circumvent the risks, conditional gene manipulation developed in animal models allows for temporal control over the expression of a transgene. Using the MerCreMer system, in particular, Cre recombinase is fused to mutant estrogen-receptor ligand-binding domains on either side (MerCreMer). This mutant estrogen receptor is insensitive to estrogen, but sensitive to TAM, an estrogen antagonist used to control the expression of MerCreMer.

Typically, cardiac-specific genetic disruption is achieved through the α-myosin heavy chain (α-MHC) promoter-driven MerCreMer expression (*Mhy6*-MerCreMer). This mouse model was first developed by Sohal *et al.*^[2]^. Although this model has been widely used to temporally control gene expression at the tissue-specific level, the possible side effects of TAM and/or MerCreMer remain controversial, thus potentially limiting the application of this approach^[3]^. Here, Rouhi *et al.*^[1]^ show the effects of TAM and MerCreMer on cardiac structure and function in mice at four weeks of age, as assessed by echocardiography. Phenotypic changes, such as myocardial fibrosis, apoptosis, and DNA damage, were determined by histological examination of the myocardium and Western blotting. They found only small and transient changes in gene expression in response to TAM and MerCreMer, but no remarkable changes in cardiac structure or function^[1]^.

The original work by Sohal *et al.*^[2]^ on TAM inducible *Mhy6*-MerCreMer mice reported no adverse effects on cardiac function, but some other studies did report transient cardiac dysfunction, dysregulated energy metabolism and cardiomyopathy^[1,3–11]^, mainly attributable to MerCreMer expression and TAM dosage. A study by Koitabashi *et al.*^[7]^ showed that TAM-induced nuclear translocation of MerCreMer resulted in transient, but severe cardiomyopathy, independent of the presence of loxP gene, thus directly pointing to MerCreMer and TAM. They associated this outcome with a significant decrease in some proteins involved in cardiac bioenergetics machinery and calcium handling, but these effects were transient and found to normalize fully upon recovery. Finally, they showed that TAM may play a role in the cardiac dysfunction seen in these mice. Other groups have corroborated these findings^[4,6]^. Studies have also shown that moderate to high doses of TAM in *Mhy6*-MerCreMer mice induced DNA damage, leading to heart failure and death^[5]^. Therefore, the authors concluded that 30 μg/g body weight of TAM resulted in maximum recombination and minimum cardiac toxicity. Another study reported the presence of focal fibrosis and depressed left-ventricular function in TAM-inducible MHC-MerCreMer mice^[8]^. This condition was found to be present regardless of the transgene. Furthermore, TAM injection induced proinflammatory cytokines, such as IL-6, TNF-α, IL-1β, IFNγ and CCL2. Markers of cardiac hypertrophy, such as ANF, BNP and COL3A1, were also found to be increased in these mice. These results prompted the authors to highlight the need to include age-matched TAM-injected MerCreMer mice as controls. In contrast, a recent work by Heinen *et al.*^[3]^ showed that using 4-hydroxyTAM (OH-Tam), a metabolite of TAM, did not induce adverse cardiotoxicity in *Mhy6*-MerCreMer mice.

In this study, Rouhi *et al.*^[1]^ studied changes in the transcriptome profile of cardiac myocytes in response to TAM and MerCreMer. TAM (30 mg/kg/day) was injected subcutaneously for five consecutive days from day 14 after birth. This time point was chosen as cardiomyocytes completely stop proliferating by this age. The dose was chosen for its reported minimal cardiac toxicity^[6,7]^. The authors performed cardiac function, myocardium histology, and mRNA expression (RNA-seq) studies 14 days after the last TAM injection. No discernible changes were noted in either myocardial histology or cardiac function, only small changes in mRNA expression in response to interferon and tumor protein 53 (TP53) pathways [Figure 1]. No significant changes were reported in the mRNA expression of cardiomyocyte transcriptome in response to TAM and MerCreMer at six months of age, suggesting that the observed mild changes in mRNA expression were transient.

From the above discussion, it will be recalled that the use of MerCreMer mice, temporally controlled by TAM, was developed to circumvent the risks associated with conventional CreLoxP gene disruption. At the same time, however, the possible side effects of TAM and/or MerCreMer remain controversial with conflicting studies. The authors emphasized the use of *Mhy6*-MerCreMer mice treated with TAM as a control, comparing to studying the gene of interest. However, the effects of TAM injection and activation of MerCreMer on cardiac function and myocardial histology at later stages, i.e., after four weeks (period chosen in this study), were not addressed. Therefore, in view of cardiac pathology, it will be necessary to conduct a time-course study of changes in the cardiac transcriptome between four weeks up to six months of age to determine the duration of changes in mRNA expression. We expect that the authors will address these limitations in future studies.

## Figures and Tables

**Figure 1.**
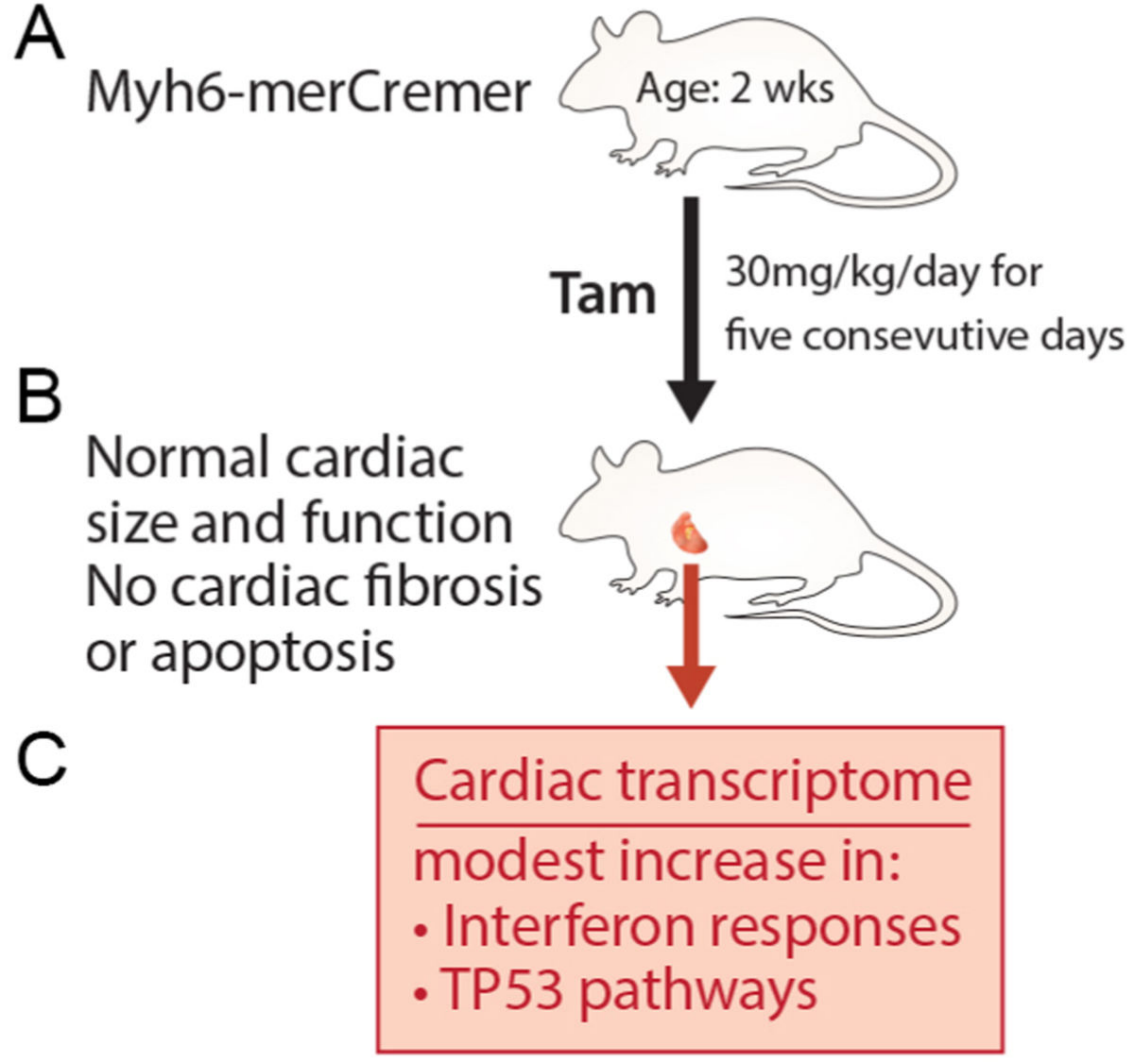
Effects of tamoxifen and MerCreMer on cardiac function, phenotype and transcriptome. Tamoxifen inducible MerCreMer expression did not cause changes in cardiac function and histology in mice at four weeks of age. Nonetheless, small, but transient changes in cardiac transcriptome in response to tamoxifen and MerCreMer expression were observed. (A) Tamoxifen was injected at 14 days to *Myh6*-MerCreMer mice. Wild-type mice lacking *Myh6*-MerCreMer were used as controls. (B) Cardiac function was assessed by echocardiography at four weeks of age prior to the sacrifice of mice. Hearts were harvested to determine cardiac fibrosis, apoptosis and DNA damage. Cardiac transcriptome profile was also assessed in the hearts of these mice. (C) Modest, but transient increases in interferon response and TP53 pathway were observed.
